# Differential Influence of Environmental Factors on Malaria Due to Vector Control Interventions in Uganda

**DOI:** 10.3390/ijerph20227042

**Published:** 2023-11-09

**Authors:** Margaux L. Sadoine, Audrey Smargiassi, Ying Liu, Philippe Gachon, Michel Fournier, Guillaume Dueymes, Jane Frances Namuganga, Grant Dorsey, Bouchra Nasri, Kate Zinszer

**Affiliations:** 1 Department of Social and Preventive Medicine, School of Public Health, Université de Montréal, Montreal, QC H3N 1X9, Canada; 2 Center for Public Health Research, Université de Montréal, Montreal, QC H3N 1X9, Canada; 3 Department of Environmental and Occupational Health, School of Public Health, Université de Montréal, Montreal, QC H3T 1A8, Canada; 4 ESCER (Étude et Simulation du Climat à l’Échelle Régionale) Centre, Université du Québec à Montréal, Montreal, QC H2L 2C4, Canada; 5 Montreal Regional Department of Public Health, Montreal, QC H2L 1M3, Canada; 6 Infectious Diseases Research Collaboration, Kampala P.O. Box 22418, Uganda; 7 Department of Medicine, University of California San Francisco, San Francisco, CA 94143, USA

**Keywords:** malaria, prevention, control, indoor residual spraying, bednets, environment, epidemiology

## Abstract

Background: Few studies have explored how vector control interventions may modify associations between environmental factors and malaria. Methods: We used weekly malaria cases reported from six public health facilities in Uganda. Environmental variables (temperature, rainfall, humidity, and vegetation) were extracted from remote sensing sources. The non-linearity of environmental variables was investigated, and negative binomial regression models were used to explore the influence of indoor residual spraying (IRS) and long-lasting insecticidal nets (LLINs) on associations between environmental factors and malaria incident cases for each site as well as pooled across the facilities, with or without considering the interaction between environmental variables and vector control interventions. Results: An average of 73.3 weekly malaria cases per site (range: 0–597) occurred between 2010 and 2018. From the pooled model, malaria risk related to environmental variables was reduced by about 35% with LLINs and 63% with IRS. Significant interactions were observed between some environmental variables and vector control interventions. There was site-specific variability in the shape of the environment–malaria risk relationship and in the influence of interventions (6 to 72% reduction in cases with LLINs and 43 to 74% with IRS). Conclusion: The influence of vector control interventions on the malaria–environment relationship need to be considered at a local scale in order to efficiently guide control programs.

## 1. Introduction

Despite significant progress made over the past 20 years, malaria remains a public health challenge worldwide, having resulted in over 247 million cases and an estimated 619,000 deaths in 2021 [1]. The control and elimination of malaria relies primarily on insecticide-treated nets (ITNs), prompt treatment with artemisinin-based combination therapies, and indoor residual spraying of insecticide (IRS). Accurate and timely surveillance can also constitute an effective tool in disease burden monitoring and intervention evaluation to guide public health policy. In 2006, an enhanced health facility-based malaria surveillance system was established in Uganda to provide high-quality data at sentinel sites, through the electronic collection of laboratory-confirmed cases of malaria. In Uganda, malaria is highly endemic, and more than 12.6 million cases were confirmed in 2021 [1]. It was the first country to introduce the mass distribution of long-lasting insecticidal nets (LLINs), which began in 2013. Since then, similar campaigns have been implemented every 3–4 years, including in 2017–2018 and most recently in 2020–2021 [2]. Uganda is one of the countries with the highest LLIN coverage, with 83% of households reportedly owning at least one LLIN in 2018, compared to 16% in 2006 [3]. Uganda has also used IRS since 2006, which is targeted to selected high-burden areas.

The environment plays a critical role in malaria transmission. Both the development and survival of the mosquito and the parasite rely on a certain threshold of rainfall, temperature, vegetation, and humidity [4,5]. The link between these factors and malaria has been widely studied, although few studies have analyzed the combined effects of environmental factors and malaria control interventions in statistical modelling [6]. Furthermore, there has not been much work exploring the differential influence of the environment with malaria control interventions, yet this information could inform control programs. Chaves et al. [7] demonstrated a reduced average effect of temperature on *P. falciparum* transmission after an ITN distribution campaign, while Carrasco-Escobar et al. [8] showed a time-varying change in slope in the dose–response effect of evapotranspiration, precipitation, and minimum temperature on malaria incidence during and after community interventions (health workers training, LLIN distribution, and community education), leading to reduced incidence rates of malaria.

The objective of this study was to investigate the influence of vector control interventions (IRS and LLINs) on associations between various environmental factors (rainfall, humidity, temperatures, and enhanced vegetation index) and malaria incident cases, using data from six public health facilities in Uganda.

## 2. Materials and Methods

### 2.1. Study Sites

This study used data from six malaria reference centers (MRCs) that are part of a health facility-based malaria surveillance program—the Uganda Malaria Surveillance Program (UMSP). UMSP began in 2006 in collaboration with the Uganda National Malaria Control Division at six sentinel sites across the county, reflective of the diversity of Uganda’s malaria transmission intensity [9]. These MRCs are high-volume level III/IV public health facilities with functional laboratories and are part of a larger MRC network that was expanded from 2014 to 2020 to include over 70 sites across the country. Each MRC captures individual-level data for all patients presenting to the outpatient department [9]. The captured information includes the following: socio-demographic variables including age and sex; village of residence; history of fever; whether malaria was suspected; whether laboratory testing was performed if malaria was suspected; what type of laboratory test was performed (rapid diagnostic test (RDT) or malaria microscopy); test results; diagnoses given; and treatments prescribed. This information is collected using a standardized register—HMIS 002: Outpatient register [9]. Initially, a data manager at each site was employed on a full-time basis by UMSP to enter data electronically using Epi Info 3.5.1 (Centers for Disease Control and Prevention, Atlanta, GA, USA). However, data entry was transitioned to health information assistants employed by the government at each facility.

**Figure 1:**
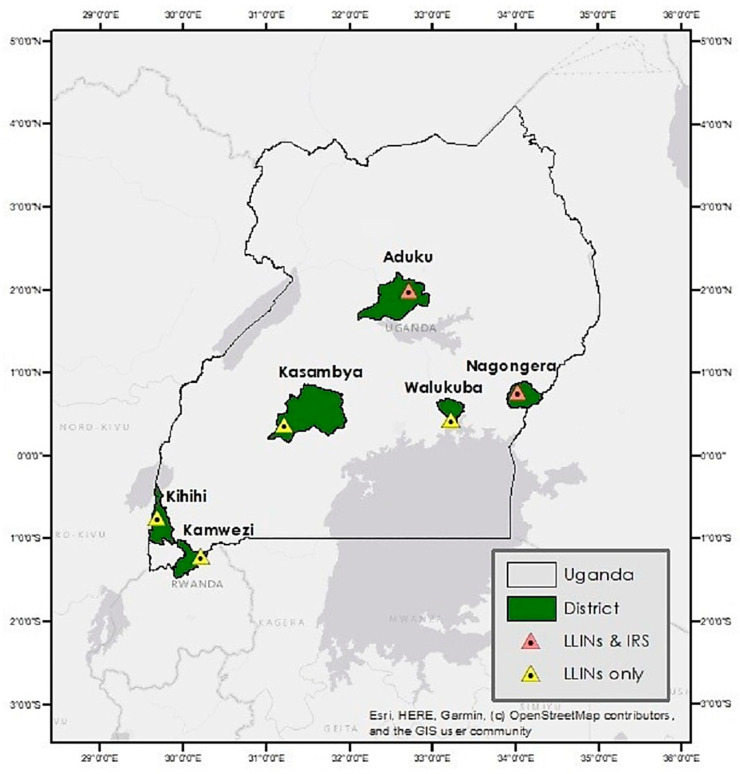
The distribution of study districts and malaria reference centers categorized by control activities.

To ensure that data collection procedures between sites were the same over time, only the original MRCs of the UMSP program were included in our study. These correspond to Aduku health center level IV (HCIV) in Kwania district, Kamwezi HCIV in Rukiga district, Kihihi HCIV in Kanungu district, Kasambya health center level III (HCIII) in Mubende district, Nagongera HCIV in Tororo district, and Walukuba HCIV in Jinja district. The locations of the districts are shown in Figure 1. Daily data provided by UMSP were converted to a weekly resolution for the period from 2010 to 2018 given the large number of zeros reported by some sites.

### 2.2. Vector Control Interventions

In Uganda, vector control efforts for malaria are focused on use of LLINs and IRS [10]. In 2013–2014, the Ministry of Health launched its first universal LLIN coverage campaign, through which LLINs were provided free of charge and targeting at least one LLIN for every 2 residents to over 90% of households [11]. This was followed by a second universal campaign in 2017/2018, which resulted in 26.5 million nets distributed, achieving a coverage of 95% [10]. The distribution dates for the first campaign were as follows: November 2013 in the districts of Mubende, Tororo, and Jinja; in May 2014 in the districts of Kwania, Kanungu, and Rukiga. The second mass distribution campaign was conducted in February 2017 in Kwania district, May 2017 in Tororo and Jinja districts, June 2017 in Kanungu and Rukiga districts, and November 2017 in Mubende district. We considered the period of LLIN coverage to begin on the first of the month following LLIN distribution and 2 years of nets’ survival. Net survivorship in African countries ranges between 6 months and 4 years, with 25 to 44% of nets unusable after 24 months and an insecticidal bio-efficacy of less than 3 years [12,13,14,15,16].

**Figure 2:**
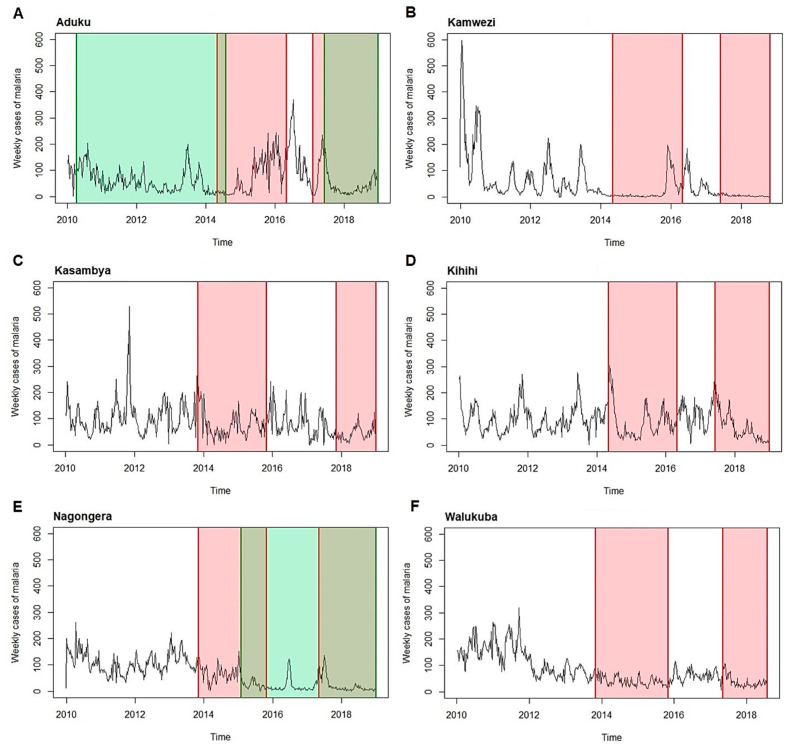
Time series of malaria weekly cases and interventions (LLIN in red, IRS in green) for 2010–2018 at each malaria reference center ((**A**): Aduku health center; (**B**): Kamwezi health center; (**C**): Kasambya health center; (**D**): Kihihi health center; (**E**): Nagongera health center; (**F**): Walukuba health center).

Indoor residual spraying (IRS) began in Uganda in the late 1950s and early 1960s with pilot projects for malaria eradication, and since then, only targeted campaigns have been conducted [17]. In 2006, Uganda expanded IRS to districts with high malaria endemicity, starting with a single round in the south-western district of Kanungu. The program was then shifted to ten high-burden districts in the north in 2007–2009. Kwania and Tororo were among the districts to receive IRS. In Kwania, one IRS round with the pyrethroid alpha-cypermethrin was implemented in March 2010, followed by nine rounds of the carbamate bendiocarb approximately every 6 months, the last of which was conducted in May 2014. After three years without IRS, one round of IRS with Actellic was implemented in May 2017. Tororo received six rounds of IRS starting in December 2014, which began with three rounds with bendiocarb sprayed approximately every 6 months, followed by three with Actellic sprayed approximately 12 months apart, with the last round included in this analysis conducted in July 2018. We considered IRS coverage from the start date of the month following its implementation up to the effectiveness of the final round. Based on a previous study [17], the effectiveness of the last bendiocarb spray round in May 2014 was estimated to have lasted 4 months after its application, and that of Actellic to have lasted 23 months. The effectiveness of Actellic was therefore considered up to December 2018. The periods considered for the interventions are shown in Figure 2 and summarized in Supplementary Table S1.

### 2.3. Environmental Data

Data on environmental factors for 2010–2018 were obtained from remote sensing sources. The Africa Rainfall Climatology Version 2 (NOAA NCEP CPC FEWS Africa DAILY ARC2 daily, available from https://iridl.ldeo.columbia.edu/, last accessed on 26 May 2020) [18] was used to obtain daily precipitation data (mm/day) for Uganda, at a 0.1°-by-0.1° horizontal resolution. The hourly near-surface air temperature, the hourly maximum and minimum near-surface air temperature, and the hourly near-surface specific humidity dataset for Uganda derived from the ERA5 re-analysis [19] at a 0.1°-by-0.1° horizontal resolution were used. Data were retrieved from the Copernicus Climate Change Service (C3S, available from https://climate.copernicus.eu/, last accessed on 30 March 2021). The gridded hourly datasets were further aggregated into daily averages.

The 16-day Enhanced Vegetation Index (EVI) dataset with a spatial resolution of 0.005° by 0.005° for Uganda was extracted from the Moderate Resolution Imaging Spectroradiometer vegetation indices products (MOS13A1 v006) [20]. This dataset was available from the U.S. Geological Survey (USGS: https://modis.gsfc.nasa.gov/data/, last accessed on 30 March 2021). The EVI imagery products collected between January 1 and January 10 of each year between 2010 and 2018 were used to represent the vegetation coverage for the dry season, and between June 10 and June 26 for the rainy seasons.

The daily average of meteorological variables (mean, maximum, and minimum temperature, humidity, and precipitation) and EVI for the six districts (i.e., Kwania, Jinja, Rukiga, Kanungu, Mubende, and Tororo) was produced from the above gridded meteorological and vegetation index products. Daily meteorological variables were then averaged over one to four months before each week of malaria counts at health facilities to consider the lag between the climatic suitability for malaria transmission and malaria onset. Cumulative rainfall (mm) for the same period was calculated.

### 2.4. Socio-Economic Data

Malaria is known to affect more those who are economically disadvantaged and is also a source of poverty [21]. Children from low-income communities have twice the risk of contracting malaria compared with children from high-income communities [22]. Therefore, we controlled associations with malaria for the average monthly household income at the sub-regional level. It corresponded to the sources of income received in cash and in-kind earnings [23]. Data were retrieved from Uganda national household surveys conducted in 2012–2013 and 2016–2017 by the Ugandan Bureau of Statistics [23,24].

### 2.5. Statistical Analysis

Potential outliers with regard to age were identified in the dataset (many people over 100 years old); therefore, patients over 70 years old (1.42%) and those with missing data on sex (0.03%) were excluded from the analyses. Spearman rank correlation analysis [25] was conducted to examine the correlation within meteorological variables. Mean daily temperature was highly correlated with maximum and minimum daily temperatures (>0.8); therefore, only maximum and minimum temperatures were considered in the subsequent analyses.

We used a general linear model (GLM) based on a negative binomial distribution to analyze the influence of interventions on the environment–malaria relationship in a pooled model, combining the six MRCs (model 1); site-specific GLMs were also created. A GLM can be used when the response variable is not distributed normally and consists of non-negative integers [26]. A GLM is made up of a linear predictor that can be written as

$$y_{i}=\beta_{0}+\beta_{1}x_{1i}+\ldots +\beta_{p}x_{p}+\varepsilon_{i}$$

where the response yi, i=1,2,…,n is modelled using a linear function of explanatory variables, plus an error term. The negative binomial distribution is used to deal with overdispersion in count data [27]. The expected value of the response is given as

$$Y=exp(y_{i})$$

In our study, the dependent variable was the number of weekly confirmed malaria cases, with a malaria case defined as positive malaria diagnostic test results via microscopy or RDT. Models included the environmental variables (maximum and minimum temperature, rainfall, humidity, and EVI), vector control interventions (yes/no LLINs and IRS), and the average monthly income of households. Sites were included in models as fixed effects. The model’s offset was represented by the weekly number of visits to each MRC, as we assumed that the number of malaria diagnoses can vary depending on the number of visits at the clinics and on the transmission seasonality. Therefore, we modelled the number of positive malaria cases among clinic visits.

Prior to fitting the models, the nonlinear relationships between environmental variables and malaria were investigated and considered using natural cubic splines with three knots placed at the 10th, 50th, and 90th percentiles. Model selection was based on the Akaike Information Criterion (AIC) [28], and model diagnostics were performed to verify the model’s fit, including Pearson and deviance residuals. The presence of residual autocorrelation was assessed through plotting the residuals according to the weeks of observations.

Finally, an additional pooled model was created (model 2) to investigate the influence of interactions between environmental variables and vector control interventions. Relevant interactions were chosen based on the LOWESS plots and the likelihood ratio test. We adopted a liberal approach to selecting interactions, in which the nested model had no interaction and was compared to the main effect model with one term of interaction at a time. A description of the final models used for analysis is presented in Table S2.

The analyses were performed using R program version 3.6.3. Models were run using the glm.nb function from the MASS package [29], nonlinear relationships were analyzed with the mgcv package [30], and the predictive margins at the mean of environmental variables at each site were produced with the ggeffects package [31]. We calculated the total predicted cases for each pool model from the predict function of the car package [32]. We also calculated the predicted cases for each site-specific model and compared their distribution to those from the pooled model without interaction.

## 3. Results

### 3.1. Characteristics of the Study Sites

A total of 204,252 laboratory-confirmed malaria cases were identified between 2010 and 2018, with an average of 73.3 weekly cases (range: 0–597). Kihihi reported the highest number of weekly cases (92.4 on average), and Kamwezi reported the lowest (47.2 on average) (Table 1). The average weekly number of visits between the sites was 425, and the highest mean was recorded in Walukuba (645 weekly visits, on average). The distribution of environmental variables averaged over 3 months (corresponding to the best average period selected for final pooled models) is presented in Table 2. Nagongera experienced the most precipitation, with an average of 363 mm for 3 months, while Kamwezi received the least amount of precipitation (a mean of 189 mm over 3 months). Kamwezi had the lowest temperature (3-month average: 14.5 °C; range: 13.7 °C–15.7 °C), while maximum temperatures were the highest in Aduku (3-month average: 28.7 °C; range: 26.8 °C–32.7 °C). The distributions of environmental factors for other averaging periods are presented in Supplementary Table S3.

### 3.2. Influence of Environmental Conditions and Intervention in the Pooled Model 1 without Interaction

The comparison between the different exposure periods (1- to 4-month averages) showed that the smallest AIC was obtained for the environmental variables averaged over a 3-month period. The regression coefficients of the final model with environmental variables averaged over a 3-month period are presented in Table S4.

**Figure 3:**
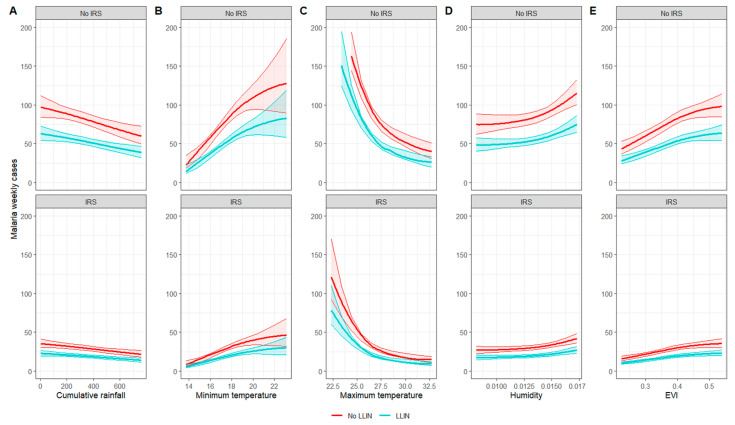
Predictive margins at the mean for each environmental variable for the pooled model without interaction ((**A**): cumulative rainfall over 3 months; (**B**): 3-month averaged minimum temperature; (**C**): 3-month averaged maximum temperature; (**D**): 3-month averaged humidity; (**E**): 3-month averaged EVI).

Predictive margins at the mean, corresponding to the predicted counts of weekly malaria cases according to each environmental variable when all other covariates were held constant at their mean, are presented in Figure 3 and Supplementary Table S5. The results suggested that malaria risk increases as minimum temperature, humidity, and EVI increase, while the risk decreased as rainfall and maximum temperature increased. The predictive margins of environmental variables on malaria risks were reduced by 35% with LLIN and approximatively 64% with IRS (Supplementary Table S5).

### 3.3. Influence of Environmental Conditions and Interventions When Considering Interactions

LOWESS plots of the relationship between malaria incident cases and each environmental variable, in the presence or absence of vector control intervention, were produced in order to identify potential interactions. The results, presented in Figures S1 and S2, did not clearly show the presence of interactions. Based on the likelihood ratio test, significant interactions were identified between IRS and minimum temperature/humidity/EVI, while all interactions between LLINs and environmental variables were statistically significant, with the exception of humidity.

To compare the two pooled models (i.e., with and without interaction), the AICs and the total number of predicted cases (sum of the weekly predicted cases over the period 2010–2018) and the predictive margins at the mean at each quantile for each environmental variable were calculated. The predictive margins at the mean are presented in Figure 2 and Table S6.

**Figure 4:**
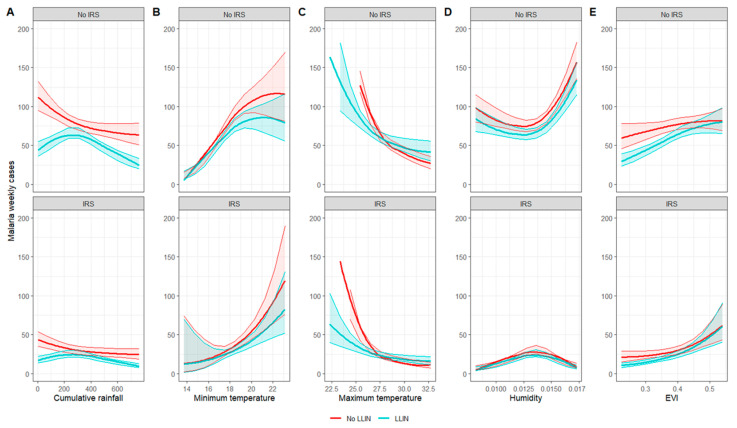
Predictive margins at the mean for each environmental variable for the pooled model with interactions between environmental variables and interventions ((**A**): cumulative rainfall over 3 months; (**B**): 3-month averaged minimum temperature; (**C**): 3-month averaged maximum temperature; (**D**): 3-month averaged humidity; (**E**): 3-month averaged EVI).

The addition of interactions in the pooled model led to a large drop in the AIC from 26,986 for model 1 (without interaction) to 26,627 for model 2 (with interaction). Results from the predictive margins’ plots suggest that the influence of interventions was not constant across the range of environmental variables. A less significant reduction in the predictive margins of precipitation by LLINs was observed between 200 and 400 mm of rain (−15% of cases at 268 mm of rain versus −60% of malaria cases at 5 mm—Table S6 and Figure 4). The reduction in the predicted cases by IRS in relation to minimum temperatures was greater, between 16 °C and 20 °C of minimum temperature, and beyond 20 °C with the LLINs. LLINs only reduced malaria predicted cases below 27 °C of maximum temperature.

Although our results highlighted interaction effects between environmental variables and the interventions, the number of total predicted cases was relatively similar between the two models: 198,770 cases for model 1 without interaction and 199,211 for model 2 with interactions, compared to 204,252 observed cases.

### 3.4. Effect of Environmental Characteristics and Intervention at Each Site

Models developed separately at each site differed from the pooled model 1. Contrary to the pool models that were developed with environmental variables averaged over 3 months, smaller AICs were obtained for site-specific models with environmental variables averaged over 4 months for Aduku, Kamwezi, Kasambya, Nagongera and Walukuba, while for Kihihi, the best AIC was for a 2-month average. The predictive margins at the mean for each environmental variable with and without intervention for each MRC are presented in Supplementary Figure S3. LLIN induced a reduction in malaria cases in all regions but Nagongera, ranging from 6 to 72% (Supplementary Tables S7–S12). IRS reduced more malaria cases in Nagongera (−74% predicted cases—Table S11) than in Aduku (−43% predicted cases—Table S7).

The comparison of the weekly malaria predictions distribution at each site from the pooled model without interaction and the site-specific models is presented in Figure S4. The results suggest that the pooled model provides similar predictions of weekly malaria cases as the site-specific models.

## 4. Discussion

We analyzed health facility-based surveillance data to examine the influence of environmental factors on malaria incident cases in the presence or absence of vector control interventions as well as examining interactions and estimations using pooled and site-specific models. We found that malaria risk was reduced more with IRS compared to LLINs. Certain interaction effects were observed between the interventions and some environmental variables. However, the predictions between the pooled models with and without interactions were relatively similar. Finally, at the site level, the effects of the environment on the risk of malaria were very heterogeneous, and interventions reduced the environmental influence in most regions.

Malaria vector control interventions using chemical insecticides have proven to be highly effective at reducing the disease incidence and mortality [33]. A meta-regression on LLIN use in Asia and Africa demonstrated a decrease in malaria prevalence of 56% (OR = 0.44, 95% CI: 0.41–0.48) [34], while some country-specific studies have showed a lower risk of malaria with LLIN ownership in Benin (IRR = 0.6, 95% CI: 0.37–0.99) [35], and LLIN use in Uganda (aRR = 0.15, 95% CI: 0.11–0.22 to aRR = 0.87, 95% CI: 0.70–1.09) [3]. The benefits of IRS have also been demonstrated, with a reduction in malaria prevalence of 62% (RR = 0.38, 95% CI: 0.31–0.46) [36] and a significant protective effectiveness in several studies conducted in Africa (median 28.5%, IQR 8.8–47.3%) [37]. Although the effectiveness of these two types of interventions has been demonstrated in several contexts, few have looked at the modification of environmental influence on malaria in the presence of antivectorial interventions. The results of the pooled model 1 confirmed the protective effects of IRS and LLIN on malaria risk, and pooled model 2 highlighted their influence in modifying the effects of environmental determinants on malaria risk.

Malaria transmission is a complex phenomenon, and multiple factors can impact the effectiveness of interventions, such as the type of insecticide and the environmental characteristics in which it is used. Environmental conditions, particularly temperature, have been found to influence the effectiveness of insecticides against Anopheles mosquitoes [38,39,40,41]. Some insecticides have a potential for toxicity, which increases with increasing temperatures (such as carbamates), or with decreasing temperatures, as is the case for pyrethroids and DDT [39,42]. Therefore, analyzing the possible effects of interactions between environmental factors and control interventions is essential to improve our understanding of this complexity. However, the effect of interventions under variable environmental conditions has been the subject of little work, and to our knowledge, only one other study has examined statistical interactions between interventions and meteorological factors, showing a variable influence of LLINs depending on the level of ambient temperature and normalized difference vegetation index [43].

In our study, the most notable interaction effects were observed between precipitation and LLINs, maximum temperatures and LLINs, and minimum temperatures and IRS. However, the total predicted cases from the model with interaction differ little from the model with no interaction, suggesting that considering interactions at the pooled scale did not have a very significant added value. Nonetheless, given the subnational variability in the effects of environmental factors on malaria and of interventions on malaria’s environmental determinants, it may be relevant to analyze finer-scale interactions to improve the understanding of interventions’ effectiveness. In our study, it was not possible to analyze the interactions on a finer scale given the number of observations per site.

Importantly, although the pooled models provide a broader understanding of the impacts of the different determinants of malaria and have the advantage of increasing the statistical power, this is based on an average effect between sites, which omits a more complex reality and variability. The analysis at a finer scale, i.e., on a local scale, demonstrated that there was great variability in the effect size and direction of the environment and in the influence of interventions on malaria risk. The reduction in malaria cases with LLINs was the highest in Kamwezi, relatively low in Kasambya and Aduku, and absent in Nagongera. The lack of LLIN protection at the Nagongera site has been observed in another study [44]. Malaria Indicator Surveys [45,46] showed systematic disparities in the accessibility, possession, and use of nets across Uganda, which could be related to the differences in the magnitude of LLINs’ influence on malaria risk observed in our study.

For IRS, our results showed that its effectiveness was greater in Nagongera than in Aduku. This difference could be due to differences in LLIN use [47], in entomological inoculation rates [48], and in insecticide type. Indeed, in Ethiopia, the performance of different insecticides was evaluated, and Bendiocarb-induced mosquito mortality was high during the first three rounds (>90%) and gradually decreased with subsequent rounds. By the sixth round, the mortality rate had reduced to 30% [49]. In our study, Aduku received nine successive rounds of Bendiocarb, while Nagongera only received three rounds before opting for Actellic. Switching the insecticide may have maintained a high level of IRS efficacy in Nagongera compared to Aduku.

Finally, it should be noted that although the shape of the relationships between the environmental variables and malaria cases differ between the subregions, the distributions of weekly predicted cases are similar between the subregional and the pooled models. The value of site-specific models therefore lies in the analysis of the influence of each individual determinant of malaria.

There are several limitations of this study. First, surveillance data were based on passive case detection and therefore only captured cases presenting to the sentinel health facilities. Second, our analyses were not stratified by age and sex, resulting in an imprecise portrait of malaria risk related to gender differences [50,51] and age [52]. Third, we used the number of clinic visits as the models’ offset. This implies that (i) we modeled the ratio of visits with malaria-positive diagnosis, which may not be a good proxy for a population-based incidence rate and is highly susceptible to bias according to access to care and diagnostic testing; (ii) we forced the case predictions to be proportional to the number of visits. This can be problematic in a negative binomial model because if the proportion of positive visits is high, the model can estimate a number of weekly positive visits for malaria that exceeds the total number of weekly visits. Despite these limits, the total number of clinic visits was used as the offset because it provided the most accurate and readily available estimate of the population at risk, given that population-based administration division estimates are outdated and do not necessarily reflect the catchment area of the MRC. Fourth, although we observed no clear temporal trend in the residuals (e.g., no seasonal trend), it is possible that there is a residual temporal dependency in the data (e.g., due to a correlation with cases of previous weeks); failure to consider this is a limitation of the results that could make the confidence intervals narrower, although this is unlikely to bias the estimates of association. Finally, the effects of LLINs were presented for a continuous two-year period from when the nets were distributed, but accessing or owning a net does not equate with its use [53]. Although results have shown a high rate of use of LLINs in the Ugandan population after the first universal distribution in 2013 [11], use may vary depending on the seasons [53] and the condition of the nets. Therefore, our findings regarding LLINs should be interpreted with caution.

## 5. Conclusions

The influence of environment and vector control interventions on malaria risk has been widely studied. However, there has been little work on how interventions modify the influence of the environment on malaria risk. Our study provides evidence that LLIN and IRS have the potential to reduce the influence of environmental factors on the risk of malaria. Malaria prevention and control programs should consider the influence of environmental factors in their programs and interventions, given the changing climate context.

## Figures and Tables

**Table 1:** Distribution of weekly cases and visits and average income for the period 2010–2018.

Variables	Aduku (*n*_week_ = 469)	Kamwezi (*n*_week_ = 461)	Kasambya (*n*_week_ = 470)	Kihihi (*n*_week_ = 469)	Nagongera (*n*_week_ = 470)	Walukuba (*n*_week_ = 448)	Overall (*n*_total_ = 2787)
Weekly cases of malaria							
Mean (SD)	68.9 (61.4)	47.4 (79.1)	86.0 (59.3)	92.4 (55.4)	64.7 (52.0)	80.3 (58.6)	73.3 (63.3)
Median (Min–Max)	49.0 (1–371)	18.0 (0–597)	72.0 (0–528)	85.0 (1–303)	60.0 (0–262)	62.0 (4–319)	58.0 (0–597)
Weekly visit numbers							
Mean (SD)	393 (122)	396 (175)	310 (116)	387 (121)	429 (114)	645 (148)	425 (169)
Median (Min–Max)	381 (86–799)	358 (143–1240)	299 (12–783)	381 (2–717)	425 (10–813)	653 (151–1060)	399 (2–1240)
Average income (k–Ugandan shillings)							
Mean (SD)	314 (93.6)	385 (62.7)	420 (50.3)	387 (63.4)	142 (5.75)	205 (11.6)	309 (117)
Median (Min–Max)	359 (139–370)	343 (339–476)	433 (329–463)	343 (339–476)	141 (137–152)	204 (195–222)	343 (137–476)

**Table 2:** Distribution of environmental factors for 3 months, averaging periods between 2010–2018.

Environmental Variables	Aduku (*n*_week_ = 469)	Kamwezi (*n*_week_ = 461)	Kasambya (*n*_week_ = 470)	Kihihi (*n*_week_ = 469)	Nagongera (*n*_week_ = 470)	Walukuba (*n*_week_ = 448)	Overall (*n*_total_ = 2787)
Cumulative rainfall (mm)							
Mean (SD)	287 (119)	189 (98.7)	265 (99.3)	263 (112)	363 (148)	300 (118)	278 (128)
Median (Min–Max)	300 (4.67, 568)	187 (8.75, 578)	266 (46.4, 485)	257 (32.6, 575)	348 (43.7, 760)	289 (77.8, 694)	268 (4.67, 760)
Maximum temperature (°C)							
Mean (SD)	28.7 (1.38)	23.5 (0.595)	27.7 (1.03)	25.8 (0.520)	27.8 (1.08)	26.7 (0.769)	26.7 (1.94)
Median (Min–Max)	28.2 (26.8, 32.7)	23.5 (22.3, 25.2)	27.5 (25.8, 30.8)	25.8 (24.6, 27.0)	27.5 (26.2, 31.1)	26.5 (25.4, 29.3)	26.9 (22.3, 32.7)
Minimum temperature (°C)							
Mean (SD)	21.1 (0.668)	14.5 (0.413)	17.7 (0.513)	16.2 (0.486)	17.5 (0.567)	19.5 (0.504)	17.8 (2.19)
Median (Min–Max)	21.1 (19.9, 23.1)	14.5 (13.7, 15.7)	17.6 (16.7, 19.5)	16.2 (15.1, 17.6)	17.5 (15.7, 19.0)	19.5 (18.4, 21.2)	17.5 (13.7, 23.1)
Humidity (kg·kg^−1^)							
Mean (SD)	0.0146 (0.000771)	0.0133 (0.00219)	0.0145 (0.000986)	0.0129 (0.00195)	0.0134 (0.00170)	0.0144 (0.000985)	0.0139 (0.00167)
Median (Min–Max)	0.0147 (0.017, 0.0161)	0.0142 (0.00830, 0.0164)	0.0146 (0.0120, 0.0164)	0.0137 (0.00817, 0.0155)	0.0136 (0.00984, 0.0173)	0.0146 (0.0119, 0.0163)	0.0143 (0.00817, 0.0173)
Enhanced vegetation index							
Mean (SD)	0.385 (0.0615)	0.414 (0.0295)	0.435 (0.0419)	0.459 (0.0348)	0.380 (0.0446)	0.371 (0.0454)	0.408 (0.0543)
Median (Min–Max)	0.391 (0.225, 0.519)]	0.408 (0.364, 0.496)	0.438 (0.297, 0.505)	0.465 (0.389, 0.539)	0.383 (0.244, 0.469)	0.379 (0.254, 0.457)	0.407 (0.225, 0.539)

## Data Availability

The datasets generated during and/or analyzed during the current study are available from the corresponding author on reasonable request.

## Supplementary Materials

The following supporting information can be downloaded at: https://www.mdpi.com/article/10.3390/ijerph20227042/s1, Table S1: Description of intervention periods; Table S2: Description of the final GLM models used for analysis; Table S3: Distribution of environmental factors between 2010–2018; Table S4: Summary of GLM negative binomial model without interaction; Table S5: Differences between predictive margins at the mean with and without interventions for the pooled negative binomial model without interaction; adjusted for average monthly income; Table S6: Differences between predictive margins at the mean with and without interventions for the pooled negative binomial model with interaction; adjusted for average monthly income; Table S7: Difference between marginal effects at the mean with and without interventions based on a site-specific negative binomial model without interaction for Aduku malaria reference center (MRC); Table S8: Difference between adjusted predictions with and without interventions based on a site-specific negative binomial model without interaction for Kamwezi MRC; Table S9: Difference between predictive margins at the mean with and without interventions based on a site-specific negative binomial model without interaction for Kasambya MRC; Table S10: Difference between predictive margins at the mean with and without interventions based on a site-specific negative binomial model without interaction for Kihihi MRC; Table S11: Difference between predictive margins at the mean with and without interventions based on a site-specific negative binomial model without interaction for Nagongera MRC; adjusted for average monthly income; Table S12: Difference between predictive margins at the mean with and without interventions based on a site-specific negative binomial model without interaction for Walukuba MRC; Figure S1: LOWESS plots of the relationship between malaria incidence and environmental variables, with and wihout LLIN; Figure S2: LOWESS plots of the relationship between malaria incidence and environmental variables, with and wihout IRS; Figure S3: Weekly predictive margins based on site specific models; Figure S4: Comparison of the weekly malaria predictions for each site from the pooled model without interaction versus the site-specific models.
