# Supplementary material for: Differential Influence of Environmental Factors on Malaria Due to Vector Control Interventions in Uganda
Source: Int J Environ Res Public Health. 2023 Nov 9;20(22):7042. doi: 10.3390/ijerph20227042 (PMC10671539; doi:10.3390/ijerph20227042)
Supplement: Supplementary file 1 [file ijerph-20-07042-s001.zip › ijerph-2543506-supplementary.pdf]

International Journal of
Environmental Research and Public Health

MDPI

Supplementary Materials

Table S1. – Description of intervention periods.

Districts (MRC site)	LLIN Coverage	Description	IRS Coverage	Description
**Kwania (Aduku)**				
	Jun 2014 – May 2016	First LLIN distribution campaign + survivorship	Apr 2010 – Sept 2014	10 continuous rounds of IRS
	Mar 2017 – Dec 2018	Second LLIN distribution campaign	Oct 2014 – May 2017	IRS withdrawal
			June 2017 – Dec 2018	1 round of IRS
**Rukiga (Kamwezi)**				
	Jun 2014 – May 2016	First LLIN distribution campaign + survivorship		
	Jul 2017 – Dec 2018	Second LLIN distribution campaign		
**Kanungu (Kihihi)**				
	Jun 2014 – May 2016	First LLIN distribution campaign + survivorship		
	Jul 2017 – Dec 2018	Second LLIN distribution campaign		
**Mubende (Kasambya)**				
	Dec 2013 – Nov 2015	First LLIN distribution campaign + survivorship		
	Dec 2017 – Dec 2018	Second LLIN distribution campaign		
**Tororo (Nagongera)**				
	Dec 2013 – Nov 2015	First LLIN distribution campaign + survivorship	Feb 2015 – Dec 2018	6 rounds of IRS
	Jun 2017 – Dec 2018	Second LLIN distribution campaign		
**Jinja (Walukuba**				
	Dec 2013 – Nov 2015	First LLIN distribution campaign		
	Jun 2017 – Dec 2018	Second LLIN distribution campaign		

Table S2. – Description of the final GLM models used for analysis.

Final models	Averaging period for environmental variables	Covariates
Pooled model 1	3 months	s(rainfall) + s(minimum temperature) + s(maximum temperature) + s(humidity) + s(EVI) + IRS + LLIN + average income + site + offset(number of visit at health centers)
Pooloed model 2	3 months	s(rainfall) + s(minimum temperature) + s(maximum temperature) + s(humidity) + s(EVI) + IRS + LLIN + average income + site + s(minimum temperature)*IRS + s(EVI)*IRS + s(humidity)*IRS + s(rainfall)*LLIN + s(minimum temperature)*LLIN + s(maximum temperature)*LLIN + s(EVI)*LLIN + offset(number of visit at health centers)
Aduku	4 months	Rainfall + s(minimum temperature) + s(maximum temperature) + humidity + s(EVI) + IRS + LLIN + average income + offset(number of visit at the health center)
Kamwezi	4 months	s(rainfall)+ minimum temperature + maximum temperature + humidity + s(EVI) + IRS + LLIN + average income + offset(number of visit at the health center)
Kasambya	4 months	s(rainfall) + minimum temperature + s(maximum temperature) + s(humidity) + s(EVI) + IRS + LLIN + average income + offset(number of visit at the health center)
Kihihi	2 months	s(rainfall) + minimum temperature + maximum temperature + humidity + s(EVI) + IRS + LLIN + average income + offset(number of visit at the health center)
Nagongera	4 months	s(rainfall) + s(minimum temperature) + maximum temperature + humidity + EVI + IRS + LLIN + average income + offset(number of visit at the health center)
Walukuba	4 months	Rainfall + s(minimum temperature) + maximum temperature + s(humidity) + EVI + IRS + LLIN + average income + offset(number of visit at the health center)

s represents the spline function ; EVI : enhanced vegetation index; IRS : indoor residual spraying; long-lasting insecticidal nets (LLIN).

Table S3. - Distribution of environmental factors between 2010 - 2018.

	**Aduku** (n_{week} **=469)**	**Kamwezi** (n_{week} **=461)**	**Kasamby a** (n_{week} **=470)**	**Kihihi** (n_{week} **=469)**	**Nagonger a** (n_{week} **=470)**	**Walukub a** (n_{week} **=448)**	**Overall** (n_{total} **=2787)**
**Cumulative rainfall (mm - 1 month)**							
Mean (SD)	95.5 (56.3)	63.0 (45.5)	88.6 (48.3)	87.8 (53.9)	121 (71.5)	99.4 (60.0)	92.6 (59.1)
Median [Min, Max]	101 [0, 267]	60.0 [0, 308]	86.5 [1.10, 227]	84.2 [0, 278]	115 [0.979, 314]	88.2 [3.24, 319]	85.8 [0, 319]
**Minimum temperatures (°C - 1 month)**							
Mean (SD)	21.1 (0.761)	14.5 (0.486)	17.7 (0.605)	16.2 (0.594)	17.5 (0.676)	19.5 (0.603)	17.8 (2.22)
Median [Min, Max]	21.1 [19.7, 23.8]	14.5 [13.1, 16.0]	17.7 [16.4, 19.8]	16.3 [14.6, 17.8]	17.5 [14.8, 19.1]	19.6 [18.0, 21.5]	17.6 [13.1, 23.8]
**Maximum temperatures (°C - 1 month)**							
Mean (SD)	28.7 (1.66)	23.5 (0.828)	27.7 (1.34)	25.8 (0.758)	27.8 (1.36)	26.7 (1.00)	26.7 (2.08)
Median [Min, Max]	28.1 [26.5, 33.5]	23.4 [21.9, 26.2]	27.3 [25.5, 32.1]	25.7 [24.0, 28.2]	27.4 [25.7, 32.2]	26.4 [25.0, 30.8]	26.8 [21.9, 33.5]
**Humidity ($kg.kg^{-1}$ – 1 month)**							
Mean (SD)	0.0146 (0.000977)	0.0133 (0.00248)	0.0145 (0.00121)	0.0129 (0.00225)	0.0134 (0.00201)	0.0144 (0.00122)	0.0138 (0.00191)
Median [Min, Max]	0.0147 [0.0116, 0.0164]	0.0145 [0.00714, 0.0166]	0.0147 [0.0112, 0.0166]	0.0139 [0.00724, 0.0158]	0.0136 [0.00873, 0.0179]	0.0146 [0.0112, 0.0168]	0.0144 [0.00714, 0.0179]
**Enhanced vegetation index (1 month)**							
Mean (SD)	0.386 (0.0825)	0.414 (0.0357)	0.435 (0.0554)	0.459 (0.0416)	0.379 (0.0590)	0.371 (0.0544)	0.408 (0.0651)
Median [Min, Max]	0.393 [0.219, 0.519]	0.407 [0.361, 0.496]	0.435 [0.279, 0.505]	0.465 [0.389, 0.539]	0.383 [0.221, 0.469]	0.394 [0.231, 0.457]	0.408 [0.219, 0.539]
**Cumulative rainfall (mm - 2 months)**							
Mean (SD)	191 (94.0)	126 (76.7)	177 (80.1)	175 (89.5)	242 (118)	200 (96.6)	185 (99.6)
Median [Min, Max]	205 [0.118, 463]	123 [0.330, 465]	181 [13.8, 337]	175 [9.63, 464]	243 [8.27, 566]	186 [13.9, 546]	177 [0.118, 566]
**Minimum temperatures (°C - 2 months)**							

Mean (SD)	21.1 (0.711)	14.5 (0.439)	17.7 (0.550)	16.2 (0.535)	17.5 (0.613)	19.5 (0.548)	17.8 (2.20)
Median [Min, Max]	21.1 [19.9, 23.6]	14.5 [13.6, 15.7]	17.6 [16.6, 19.8]	16.3 [15.0, 17.7]	17.5 [15.2, 19.0]	19.6 [18.3, 21.5]	17.6 [13.6, 23.6]
**Maximum temperatures (°C - 2 months)**							
Mean (SD)	28.7 (1.52)	23.5 (0.700)	27.7 (1.18)	25.8 (0.613)	27.8 (1.21)	26.7 (0.870)	26.7 (2.00)
Median [Min, Max]	28.2 [26.7, 33.4]	23.5 [22.2, 25.4]	27.4 [25.6, 31.1]	25.8 [24.5, 27.3]	27.4 [26.1, 31.9]	26.5 [25.2, 29.7]	26.8 [22.2, 33.4]
**Humidity (kg.kg^{-1} - 2 months)**							
Mean (SD)	0.0146 (0.000874)	0.0133 (0.00235)	0.0145 (0.00111)	0.0129 (0.00212)	0.0134 (0.00185)	0.0144 (0.00111)	0.0138 (0.00180)
Median [Min, Max]	0.0148 [0.0123, 0.0164]	0.0143 [0.00770, 0.0164]	0.0147 [0.0116, 0.0165]	0.0138 [0.00766, 0.0156]	0.0135 [0.00959, 0.0176]	0.0146 [0.0114, 0.0165]	0.0144 [0.00766, 0.0176]
**Enhanced vegetation index (2 months)**							
Mean (SD)	0.386 (0.0724)	0.414 (0.0325)	0.435 (0.0485)	0.459 (0.0382)	0.380 (0.0519)	0.371 (0.0499)	0.408 (0.0597)
Median [Min, Max]	0.392 [0.219, 0.519]	0.408 [0.361, 0.496]	0.436 [0.279, 0.505]	0.466 [0.389, 0.539]	0.383 [0.221, 0.469]	0.387 [0.231, 0.457]	0.408 [0.219, 0.539]
**Cumulative rainfall (mm - 4 months)**							
Mean (SD)	382 (132)	252 (110)	353 (103)	351 (119)	483 (159)	400 (124)	370 (143)
Median [Min, Max]	388 [60.0, 673]	260 [31.4, 648]	347 [125, 631]	342 [94.8, 659]	471 [121, 891]	385 [150, 781]	358 [31.4, 891]
**Minimum temperature (°C – 4 months)**							
Mean (SD)	21.1 (0.626)	14.5 (0.396)	17.7 (0.482)	16.2 (0.443)	17.5 (0.528)	19.5 (0.464)	17.8 (2.19)
Median [Min, Max]	21.1 [20.1, 23.0]	14.5 [13.8, 15.7]	17.6 [16.9, 19.2]	16.2 [15.2, 17.5]	17.5 [15.9, 18.9]	19.5 [18.6, 21.0]	17.5 [13.8, 23.0]
**Maximum temperature (°C – 4 months)**							
Mean (SD)	28.7 (1.25)	23.5 (0.500)	27.7 (0.881)	25.7 (0.448)	27.8 (0.961)	26.7 (0.676)	26.7 (1.89)
Median [Min, Max]	28.3 [26.8, 32.0]	23.4 [22.4, 24.9]	27.5 [26.0, 30.2]	25.8 [24.6, 26.7]	27.6 [26.2, 30.4]	26.5 [25.5, 28.8]	26.9 [22.4, 32.0]
**Humidity**							

**(kg.kg^{-1} – 4 months)**							
Mean (SD)	0.0146 (0.000670)	0.0133 (0.00199)	0.0145 (0.000866)	0.0129 (0.00176)	0.0134 (0.00154)	0.0145 (0.000869)	0.0139 (0.00154)
Median [Min, Max]	0.0146 [0.0130, 0.0158]	0.0141 [0.00891, 0.0164]	0.0146 [0.0124, 0.0161]	0.0136 [0.00892, 0.0155]	0.0136 [0.0102, 0.0170]	0.0145 [0.0122, 0.0163]	0.0143 [0.00891, 0.0170]
**Enhanced vegetation index (4 months)**							
Mean (SD)	0.385 (0.0506)	0.414 (0.0271)	0.435 (0.0358)	0.459 (0.0319)	0.380 (0.0375)	0.371 (0.0413)	0.407 (0.0495)
Median [Min, Max]	0.391 [0.257, 0.519]	0.408 [0.366, 0.496]	0.439 [0.321, 0.505]	0.466 [0.390, 0.539]	0.381 [0.273, 0.469]	0.374 [0.275, 0.457]	0.407 [0.257, 0.539]

Table S4. - Summary of GLM negative binomial model without interaction.

	**Malaria weekly cases**		
Predictors	***Incidence Rate Ratios***	*CI*	*p*
IRS [Yes]	0.37	0.33 – 0.40	**< 2.20 e^{-16}**
LLIN [Yes]	0.65	0.62 – 0.68	**< 2.20 e^{-16}**
Average income	9.99 e^{-01}	9.99 e^{-01}– 9.99 e^{-01}	**< 2.20 e^{-16}**
Rainfall (mm) - 1	1.00	0.99 – 1.00	0.104
Rainfall (mm) - 2	1.00	0.99 – 1.00	0.4339
Minimum temperature (°C) - 1	1.40	1.27 – 1.55	**1.23 e^{-10}**
Minimum temperature (°C) - 2	0.81	0.74 – 0.90	**0.000**
Maximum temperature (°C) - 1	0.70	0.64 – 0.77	**5.50 e^{-16}**
Maximum temperature(°C) - 2	1.14	1.08 – 1.21	**4.96 e^{-06}**
Humidity (kg.kg-1) - 1	6.88 e^{07}	2.28 e^{-07} – 2.28 e^{22}	0.278
Humidity (kg.kg-1) - 2	9.99 e^{14}	44.38 –2.07 e^{28}	**0.028**
EVI -1	42.8	15.63 – 117.46	**3.07 e^{-13}**
EVI - 2	0.15	0.04 – 0.48	**0.001**
MRC*			
Kamwezi	0.24	0.17 – 0.34	**< 2.20 e^{-16}**
Kasambya	1.01	0.79 – 1.29	0.934

Kihihi	0.83	0.63 – 1.09	0.175
Nagongera	0.75	0.59 – 0.95	**0.019**
Walukuba	0.28	0.24 – 0.33	**< 2.20 e^{-16}**
Observations		2787	

*Aduku is the reference

Table S5. – Differences between predictive margins at the mean with and without interventions for the pooled negative binomial model without interaction; adjusted for average monthly income.

Variable	Predicted cases			Difference in % between predictions without and with IRS	Difference in % between predictions without and with LLIN
	No IRS / no LLIN	IRS	LLIN		
**Rain (mm)**					
**Min - 4.67**	**97.11**	35.21	62.99	-63.74	-35.14
**Q1 - 185.53**	**89.51**	32.45	58.05	-63.75	-35.15
**Q2 - 268.38**	**85.71**	31.07	55.58	-63.75	-35.15
**Q3 - 357.54**	**80.86**	29.31	52.44	-63.75	-35.15
**Max - 760.02**	**59.90**	21.71	38.85	-63.76	-35.14
**Minimum temperature (°C)**					
Min – 13.74	25.80	9.35	16.73	-63.76	-35.16
Q1 - 16.22	58.31	21.14	37.81	-63.75	-35.16
Q2 - 17.54	81.62	29.59	52.93	-63.75	-35.15
Q3 - 19.51	105.89	38.39	68.67	-63.75	-35.15
Max - 23.12	128.69	46.65	83.46	-63.75	-35.15
**Maximum temperature (°C)**					
Min – 22.33	345.28	125.17	223.92	-63.75	-35.15
Q1 - 25.76	108.48	39.33	70.36	-63.74	-35.14
Q2 - 26.85	82.46	29.89	53.48	-63.75	-35.14
Q3 - 27.4	69.46	25.18	45.05	-63.75	-35.14
Max - 32.72	39.57	14.34/	25.66	-63.75	-35.15
**Humidity (kg.kg^{-1})**					
Min – 0.82 x 10^{-2}	74.06	26.85	48.03	-63.75	-35.15
Q1 - 1.31 x 10^{-2}	82.21	29.80	53.32	-63.75	-35.14
Q2 - 1.43 x 10^{-2}	87.94	31.88	57.03	-63.75	-35.15
Q3 - 1.50 x 10^{-2}	93.34	33.84	60.53	-63.75	-35.15
Max - 1.72 x 10^{-2}	115.27	41.79	74.76	-63.75	-35.14
**EVI**					
Min – 0.22	44.43	16.11	28.81	-63.74	-35.16
Q1 - 0.38	78.03	28.29	50.60	-63.74	-35.15
Q2 - 0.41	85.18	30.88	55.24	-63.75	-35.15
Q3 - 0.45	90.84	32.93	58.91	-63.75	-35.15
Max – 0.54	98.31	35.64	63.76	-63.75	-35.14

Table S6. - Differences between predictive margins at the mean with and without interventions for the pooled negative binomial model with interaction; adjusted for average monthly income.

Variable	Predicted cases			Difference in % between predictions without and with IRS	Difference in % between predictions without and with LLIN
	No IRS / no LLIN	IRS	LLIN		
**Rain (mm)**					
**Min - 4.67**	**112.25**	43.47	44.87	-61.27	-60.03
**Q1 - 185.53**	**86.70**	33.58	62.34	-61.27	-28.10
**Q2 - 268.38**	**78.70**	30.48	66.76	-61.27	-15.17
**Q3 - 357.54**	**73.86**	28.60	61.47	-61.28	-16.77
**Max - 760.02**	**63.17**	24.46	25.72	-61.28	-59.28
**Minimum temperature (°C)**					
Min – 13.74	11.05	12.38	10.58	12.04	-4.25
Q1 - 16.22	42.69	21.19	38.28	-50.36	-10.33
Q2 - 17.54	73.07	28.65	62.93	-60.79	-13.88
Q3 - 19.51	103.88	46.81	83.06	-54.94	-20.04
Max - 23.12	117.19	120.26	80.51	2.62	-31.30
**Maximum temperature (°C)**					
Min – 22.33	705.24	273.10	165.95	-61.28	-76.47
Q1 - 25.76	113.26	43.86	77.99	-61.27	-31.14
Q2 - 26.85	74.21	28.74	65.27	-61.27	-12.05
Q3 - 27.4	57.64	22.32	58.44	-61.28	1.39
Max - 32.72	26.77	10.37	40.86	-61.26	52.63
**Humidity (kg.kg^{-1})**					
Min – 0.82 x 10^{-2}	95.80	6.91	81.88	-92.79	-14.53
Q1 - 1.31 x 10^{-2}	74.85	29.54	63.97	-60.53	-14.54
Q2 - 1.43 x 10^{-2}	82.64	28.41	70.63	-65.62	-14.53
Q3 - 1.50 x 10^{-2}	94.49	23.04	80.76	-75.62	-14.53
Max - 1.72 x 10^{-2}	157.38	9.60	134.51	-93.90	-14.53
**EVI**					
Min – 0.22	60.03	20.70	30.38	-65.52	-49.39
Q1 - 0.38	75.33	27.43	59.88	-63.59	-20.51
Q2 - 0.41	78.00	30.18	66.63	-61.31	-14.58
Q3 - 0.45	79.92	36.56	72.28	-54.25	-9.56
Max – 0.54	82.11	62.07	80.45	-24.41	-2.02

Table S7. Difference between predictive margins at the mean with and without interventions based on a site-specific negative binomial model without interaction for Aduku malaria reference center (MRC); adjusted for average monthly income.

Variable	Predicted cases			Difference in % between predictions without and with IRS	Difference in % between predictions without and with LLIN
	No LLIN / no IRS	IRS	LLIN		
**Rain (mm)**					
Min - 60.02	84.06	47.45	76.54	-43.55	-8.95
Q1 - 293.96	83.17	46.95	75.73	-43.55	-8.95
Q2 - 388.40	82.81	46.75	75.40	-43.55	-8.95
Q3 - 465.95	82.51	46.58	75.13	-43.55	-8.94
Max - 672.89	81.73	46.14	74.42	-43.55	-8.94

**Minimum temperature (°C)**					
Min - 20.06	108.34	61.16	98.65	-43.55	-8.94
Q1 – 20.66	87.08	46.16	79.26	-46.99	-8.98
Q2 – 21.07	82.58	46.62	75.19	-43.55	-8.95
Q3 – 21.51	90.89	51.31	82.76	-43.55	-8.94
Max - 22.95	253.81	143.28	231.10	-43.55	-8.95
**Maximum temperature (°C)**					
Min - 26.80	54.64	30.85	49.75	-43.54	-8.95
Q1 – 27.65	70.85	40.00	64.51	-43.54	-8.95
Q2 – 28.32	80.86	45.65	73.62	-43.54	-8.95
Q3 – 29.58	76.95	43.44	70.06	-43.55	-8.95
Max - 32.03	34.50	19.48	31.42	-43.54	-8.93
**Humidity (kg.kg^{-1})**					
Min – 1.3 x 10^{-2}	108.00	60.97	98.34	-43.55	-8.94
Q1 – 1.4 x 10^{-2}	89.09	50.30	81.12	-43.54	-8.95
Q2 – 1.5 x 10^{-2}	82.59	46.62	75.20	-43.55	-8.95
Q3 - 1.5 x 10^{-2}	76.10	42.96	69.29	-43.55	-8.95
Max – 1.6 x 10^{-2}	68.54	38.69	62.41	-43.55	-8.94
**EVI**					
Min - 0.26	50.80	28.68	46.26	-43.54	-8.94
Q1 - 0.35	72.50	40.93	66.02	-43.54	-8.94
Q2 - 0.39	84.60	47.76	77.03	-43.55	-8.95
Q3 – 0.41	92.21	52.05	83.96	-43.55	-8.95
Max - 0.52	138.19	78.01	125.83	-43.55	-8.94

Table S8. - Difference between adjusted predictions with and without interventions based on a site-specific negative binomial model without interaction for Kamwezi MRC; adjusted for average monthly income.

Variable	Predicted cases		Difference in % between predictions without and with LLIN
	No LLIN	LLIN	
**Rain (mm)**			-72.01
Min - 31.38	32.87	9.20	-72.00
Q1 - 172.00	50.43	14.12	-72.00
Q2 - 259.91	53.71	15.04	-72.00
Q3 - 314.33	48.92	13.70	-71.93
Max - 648.46	6.02	1.69	
**Minimum temperature (°C)**			
Min – 13.81	20.43	5.72	-72.00
Q1 – 14.23	35.55	9.95	-72.01
Q2 – 14.50	51.31	14.36	-72.01
Q3 – 14.77	73.35	20.53	-72.01
Max – 15.67	242.98	68.02	-72.01
**Maximum temperature (°C)**			

Min – 22.37	125.22	35.06	-72.00
Q1 – 23.14	70.22	19.66	-72.00
Q2 – 23.43	56.48	15.81	-72.01
Q3 – 23.85	41.20	11.53	-72.01
Max – 24.88	19.07	5.34	-72.00
**Humidity (kg.kg^{-1})**			
Min – 0.08 x 10^{-2}	81.29	22.76	-72.00
Q1 – 1.15 x 10^{-2}	49.84	13.95	-72.01
Q2 - 1.40 x 10^{-2}	47.71	13.36	-72.00
Q3 – 1.51 x 10^{-2}	45.51	12.74	-72.01
Max – 1.64 x 10^{-2}	40.61	11.37	-72.00
**EVI**			
Min – 0.37	13.49	3.78	-71.98
Q1 – 0.40	37.97	10.63	-72.00
Q2 – 0.41	48.66	13.62	-72.01
Q3 – 0.43	63.06	17.65	-72.01
Max – 0.50	33.38	9.48	-71.60

Table S9. - Difference between predictive margins at the mean with and without interventions based on a site-specific negative binomial model without interaction for Kasambya MRC; adjusted for average monthly income.

Variable	Predicted cases		Difference in % between predictions without and with LLIN
	No LLIN	LLIN	
**Rain (mm)**			
Min – 125.37	45.71	42.95	-6.04
Q1 – 280.07	73.14	68.73	-6.03
Q2 - 346.91	84.38	79.29	-6.03
Q3 - 421.60	92.40	86.83	-6.03
Max - 630.62	93.09	87.47	-6.04
**Minimum temperature (°C)**			
Min – 16.87	84.59	79.48	-6.04
Q1 – 17.25	84.88	79.76	-6.03
Q2 – 17.60	85.15	80.01	-6.04
Q3 – 17.98	85.44	80.29	-6.03
Max – 19.21	86.39	81.18	-6.03
**Maximum temperature (°C)**			
Min – 25.96	74.97	70.45	-6.03
Q1 – 27.07	85.10	79.96	-6.04
Q2 – 27.50	85.84	80.66	-6.03
Q3 – 28.21	80.67	75.80	-6.04
Max – 30.21	48.79	45.85	-6.03
**Humidity (kg.kg^{-1})**			
Min – 1.24 x 10^{-2}	111.27	104.56	-6.03
Q1 – 1.39 x 10^{-2}	86.77	81.54	-6.03
Q2 – 1.46 x 10^{-2}	85.51	80.35	-6.03
Q3 - 1.51 x 10^{-2}	90.73	85.26	-6.03
Max - 1.61 x 10^{-2}	110.77	104.09	-6.03
**EVI**			
Min – 0.32	33.45	31.43	-6.04
Q1 – 0.42	80.13	75.30	-6.03
Q2 – 0.44	85.82	80.65	-6.02

Q3 – 0.46	85.15	80.02	-6.02
Max – 0.50	74.21	69.73	-6.04

Table S10. - Difference between predictive margins at the mean with and without interventions based on a site-specific negative binomial model without interaction for Kihihi MRC; adjusted for average monthly income.

Variable	Predicted cases		Difference in % between predictions without and with LLIN
	No LLIN	LLIN	
**Rain (mm)**			
Min – 9.63	127.22	84.05	-33.93
Q1 – 103.47	106.30	70.23	-33.93
Q2 – 175.15	94.99	62.76	-33.93
Q3 – 242.72	88.33	58.36	-33.93
Max – 463.86	80.89	53.44	-33.93
**Minimum temperature (°C)**			
Min – 14.9	49.32	32.59	-33.92
Q1 – 15.93	80.53	53.21	-33.93
Q2 – 16.26	95.88	63.35	-33.93
Q3 – 16.56	111.92	73.95	-33.93
Max – 17.67	199.72	131.95	-33.93
**Maximum temperature (°C)**			
Min – 24.50	154.29	101.94	-33.93
Q1 – 25.35	111.33	73.56	-33.93
Q2 – 25.80	93.59	61.84	-33.92
Q3 – 26.18	80.62	53.27	-33.92
Max – 27.33	51.87	34.27	-33.93
**Humidity (kg.kg^{-1})**			
Min – 0.77	128.52	84.91	-33.93
Q1 – 1.11	105.31	69.58	-33.93
Q2 – 1.4	89.75	59.30	-33.93
Q3 – 1.5	86.63	57.24	-33.93
Max – 1.6	81.22	53.66	-33.93
**EVI**			
Min – 0.39	88.27	58.32	-33.93
Q1 – 0.43	95.17	62.88	-33.93
Q2 – 0.47	90.74	59.95	-33.93
Q3 – 0.48	94.15	62.20	-33.94
Max – 0.54	69.38	45.84	-33.93

Table S11. - Difference between predictive margins at the mean with and without interventions based on a site-specific negative binomial model without interaction for Nagongera MRC; adjusted for average monthly income.

Variable	Predicted cases			Difference between predictions without and with IRS	Difference between predictions without and with LLIN
	No LLIN / no IRS	IRS	LLIN		
**Rain (mm)**					
Min – 121.41	22.05	5.74	23.67	-73.97	7.35
Q1 – 361.95	59.92	15.60	64.32	-73.97	7.34
Q2 – 470.59	79.60	20.72	85.44	-73.97	7.34
Q3 – 604.78	89.93	23.41	96.52	-73.97	7.33
Max – 891.27	68.69	17.88	73.73	-73.97	7.34
**Minimum temperature (°C)**					
Min – 15.90	178.92	46.58	192.05	-73.97	7.34
Q1 – 17.19	84.66	22.04	90.87	-73.97	7.34
Q2 – 17.48	81.35	21.19	87.35	-73.95	7.38
Q3 – 17.89	87.07	22.67	93.46	-73.96	7.34
Max – 18.94	157.52	41.01	169.08	-73.97	7.34
**Maximum temperature (°C)**					
Min – 22.18	60.13	15.65	64.54	-73.97	7.33
Q1 – 27.08	71.11	18.51	76.32	-73.97	7.33
Q2 – 27.55	77.62	20.21	83.32	-73.96	7.34
Q3 – 28.49	92.42	24.06	99.20	-73.97	7.34
Max – 30.37	131.35	34.20	140.99	-73.96	7.34
**Humidity (kg.kg^{-1})**					
Min – 1.02	80.80	21.04	86.73	-73.96	7.34
Q1 – 1.22	81.17	21.13	87.12	-73.97	7.33
Q2 – 1.36	81.41	21.20	87.39	-73.96	7.35
Q3 – 1.45	81.57	21.24	87.55	-73.96	7.33
Max – 1.70	82.02	21.35	88.04	-73.97	7.34
**EVI**					
Min – 0.27	67.68	17.62	72.64	-73.97	7.33
Q1 – 0.36	78.92	20.55	84.71	-73.96	7.34
Q2 – 0.38	81.56	21.23	87.54	-73.97	7.33
Q3 – 0.41	85.33	22.22	91.59	-73.96	7.34
Max – 0.47	94.93	24.71	101.89	-73.97	7.33

Table S12. - Difference between predictive margins at the mean with and without interventions based on a site-specific negative binomial model without interaction for Walukuba MRC; adjusted for average monthly income.

Variable	Predicted cases		Difference in % between predictions without and with LLIN
	No LLIN	LLIN	
**Rain (mm)**			
Min - 149.98	125.81	65.43	-47.99
Q1 – 308.88	104.67	54.44	-47.99
Q2 – 384.57	95.89	49.87	-47.99
Q3 – 480.53	85.80	44.63	-47.98
Max – 780.87	60.61	31.52	-48.00
**Minimum temperature (°C)**			
Min – 18.62	71.79	37.34	-47.99
Q1 – 19.20	88.24	45.89	-47.99
Q2 – 19.51	93.92	48.85	-47.99
Q3 – 19.86	94.02	48.90	-47.99
Max – 20.96	69.25	36.02	-47.99
**Maximum temperature (°C)**			
Min – 25.46	137.47	71.50	-47.99
Q1 – 26.20	109.92	57.17	-47.99
Q2 – 26.55	99.20	51.60	-47.98
Q3 – 27.15	82.76	43.05	-47.98
Max – 28.75	51.24	26.65	-47.99
**Humidity (kg.kg^{-1})**			
Min – 1.22 x 10^{-2}	44.29	23.03	-48.00
Q1 – 1.39 x 10^{-2}	81.89	42.59	-47.99
Q2 – 1.45 x 10^{-2}	96.12	49.99	-47.99
Q3 – 1.50 x 10^{-2}	104.55	54.38	-47.99
Max – 1.63 x 10^{-2}	113.59	59.08	-47.99
**EVI**			
Min – 0.27	75.48	39.26	-47.99
Q1 – 0.35	88.95	46.27	-47.98
Q2 – 0.37	94.80	49.31	-47.99
Q3 – 0.40	101.03	52.55	-47.99
Max – 0.46	114.54	59.57	-47.99

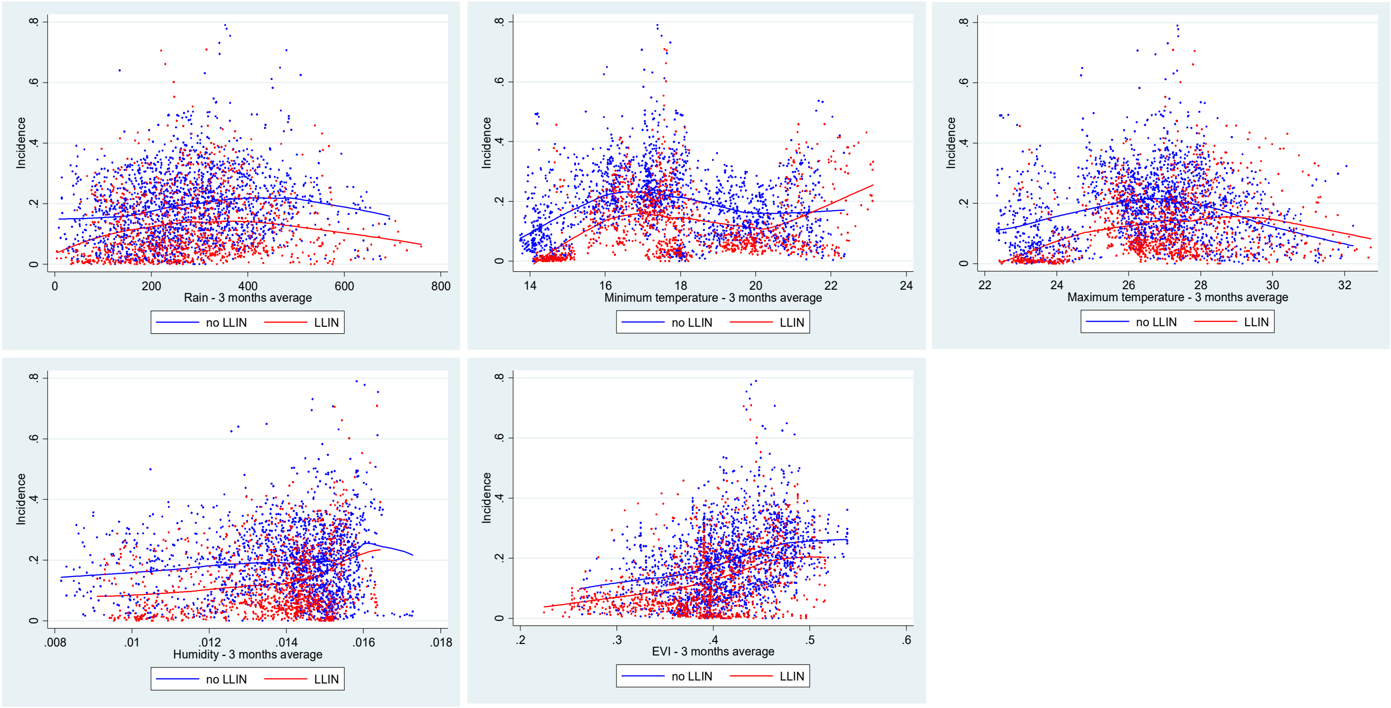

Figure S1. – LOWESS plots of the relationship between malaria incidence and environmental variables, with and wihout LLIN.

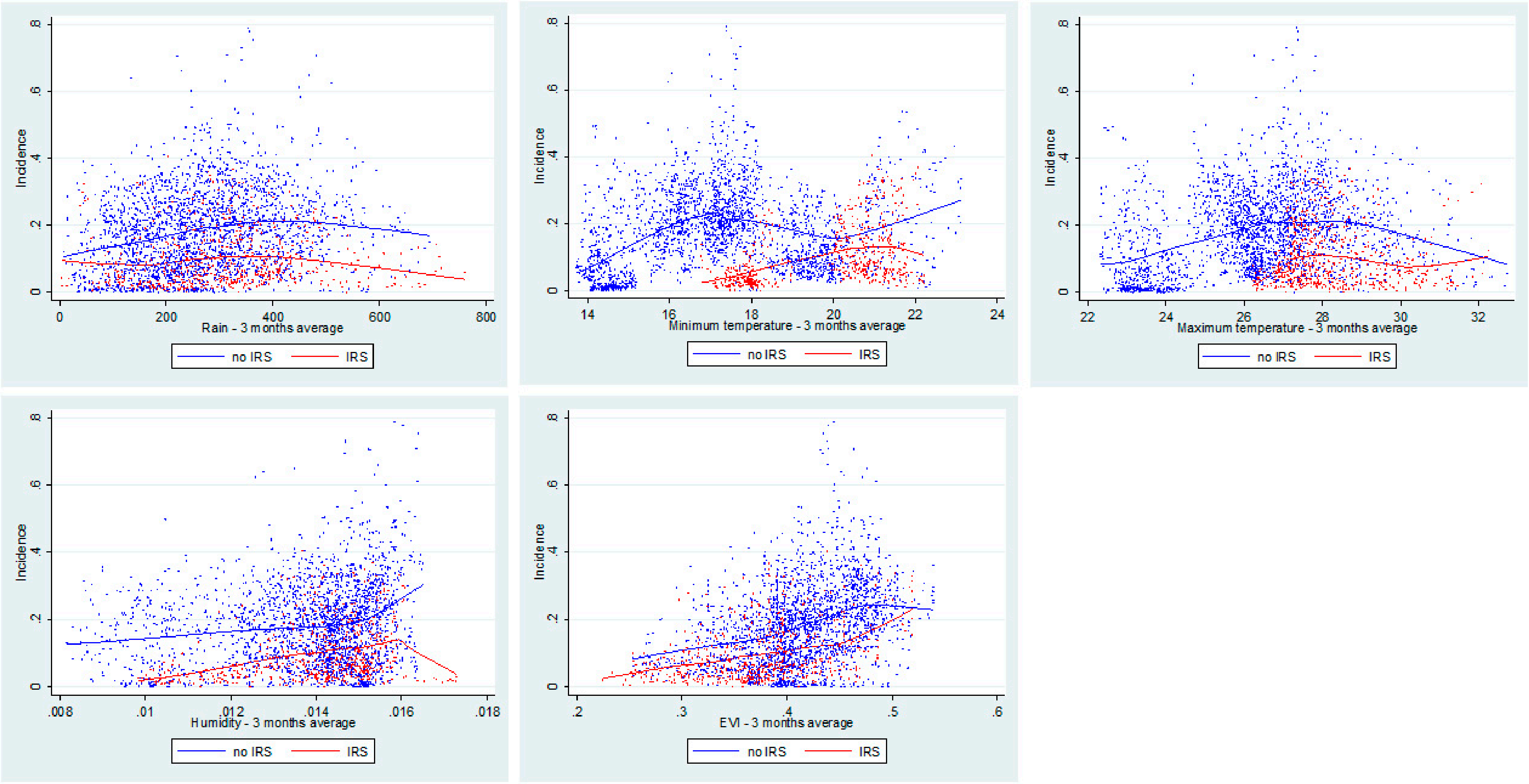

Figure S2. – LOWESS plots of the relationship between malaria incidence and environmental variables, with and wihout IRS.

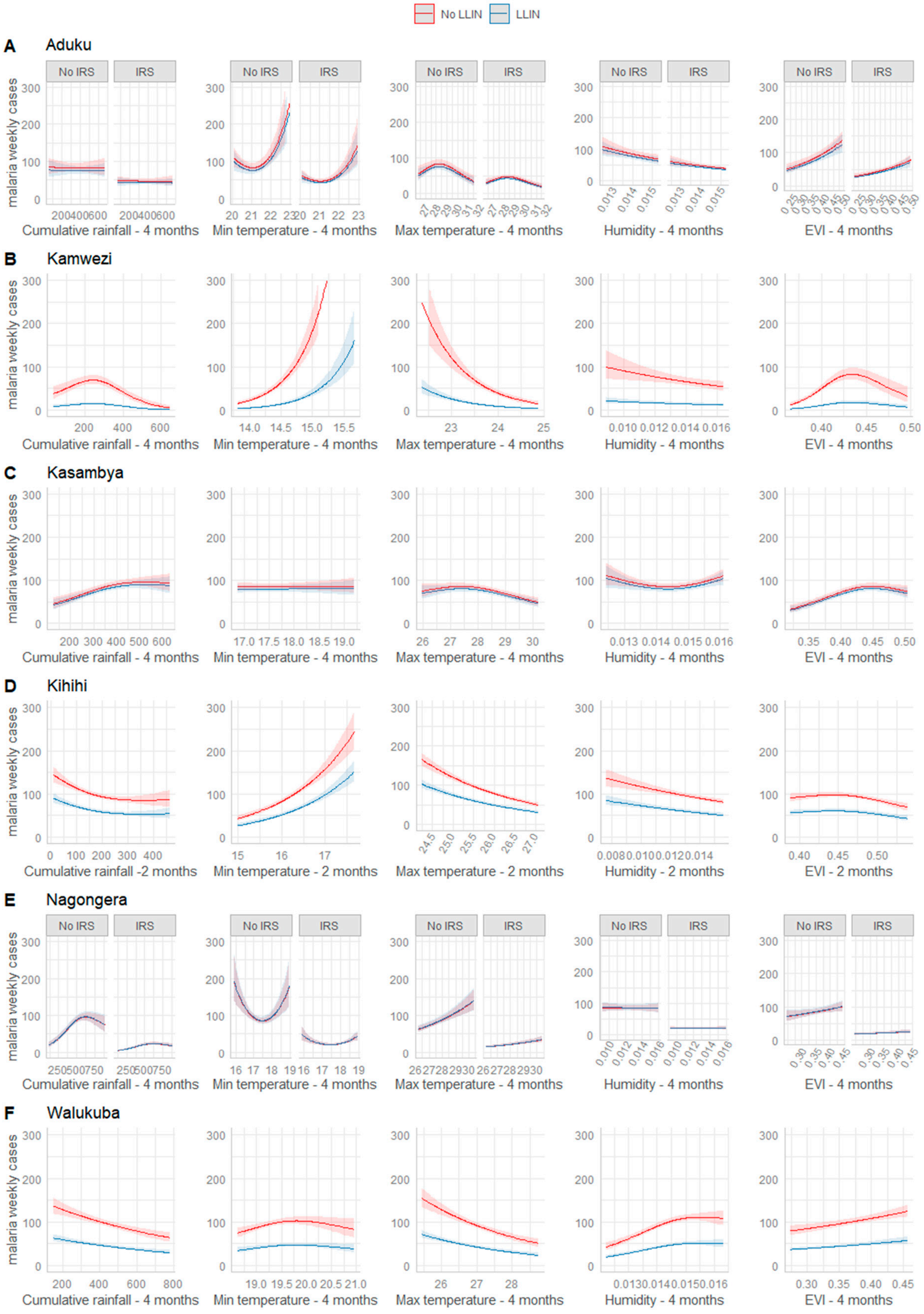

Figure S3. - Weekly predictive margins based on site specific models (A: Adkuku, B: Kamwezi, C: Kasambya, D: Kihihi, E: Nagongera, F: Walukuba); predictive margins are based on a negative binomial model without interaction,

adjusted for average monthly income.

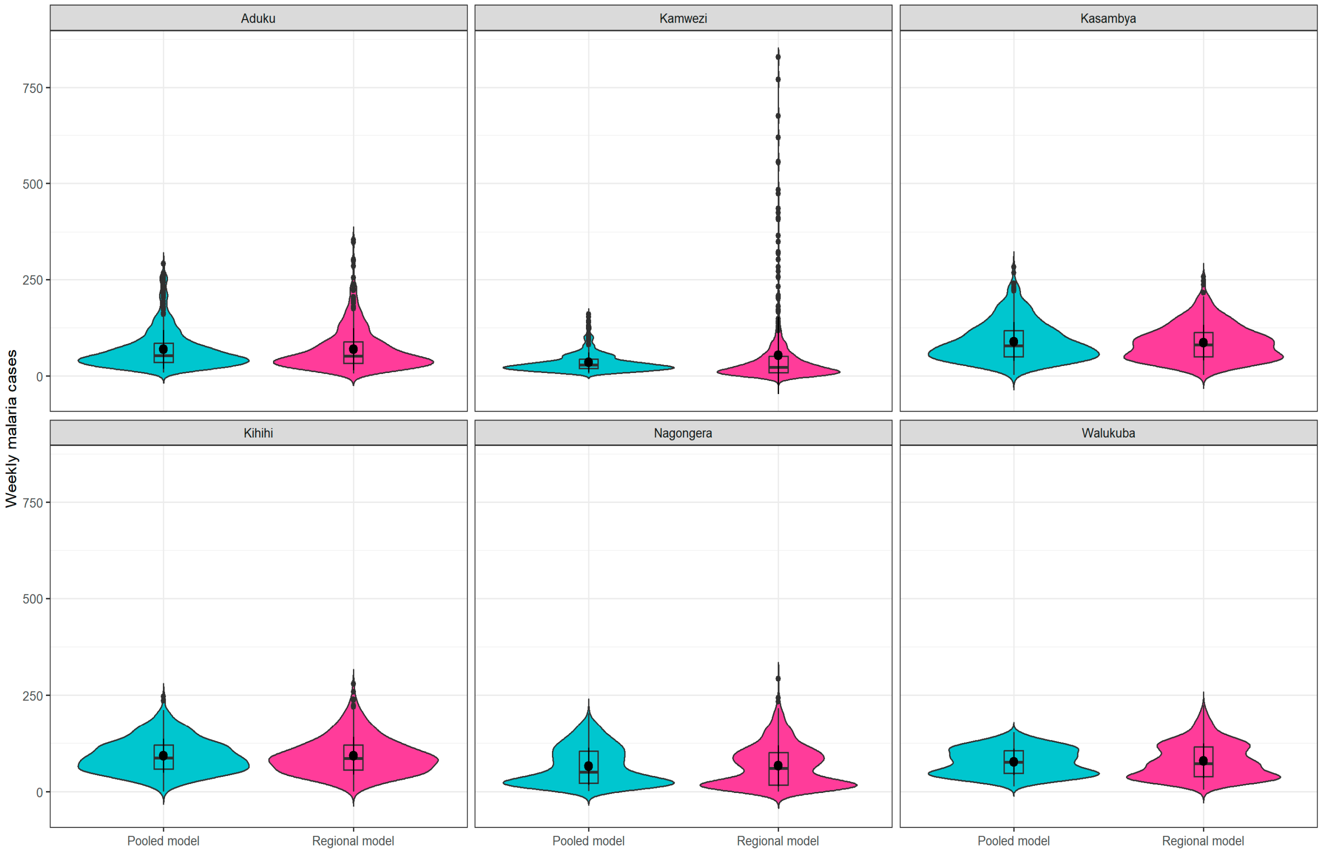

Figure S4. - Comparison of the weekly malaria predictions for each site from the pooled model without interaction versus the site-specific models; (N=53 weeks*6 regions *9 years).
